# Investigation of *ICOS, CD28* and *CD80* polymorphisms with the risk of hepatocellular carcinoma: a case–control study in eastern Chinese population

**DOI:** 10.1042/BSR20181824

**Published:** 2019-07-05

**Authors:** Jing Yang, Jiaochun Liu, Yu Chen, Weifeng Tang, Kai Bo, Yuling Sun, Jianping Chen

**Affiliations:** 1 Department of Gastroenterology, The Third Affiliated Hospital of Soochow University, Changzhou, Jiangsu Province, China; 2 Department of Gastroenterology, People’s Liberation Army 92nd Hospital, Nanping, Fujian Province,China; 3 Cancer Bio-immunotherapy Center, Fujian Cancer Hospital and Fujian Medical University Cancer Hospital, Fuzhou, Fujian Province, China; 4 Department of Medical Oncology, Fujian Cancer Hospital and Fujian Medical University Cancer Hospital, Fuzhou, Fujian Province, China; 5 Fujian Provincial Key Laboratory of Translational Cancer Medicine, Fuzhou, Fujian Province, China; 6 Department of Cardiothoracic Surgery, Affiliated People’s Hospital of Jiangsu University, Zhenjiang, Jiangsu Province, China; 7 Department of Hepatobiliary and Pancreatic Surgery, The First Affiliated Hospital of Zhengzhou University, Zhengzhou, Henan Province, China; 8 Institute of Hepatobiliary and Pancreatic Diseases, Zhengzhou University, Zhengzhou, Henan Province, China

**Keywords:** CD28, CD80, Hepatocellular carcinoma, ICOS, Polymorphism, Risk

## Abstract

Single nucleotide polymorphisms (SNPs) in immune related gene may influence the susceptibility of cancer. We selected *inducible T cell costimulator* (*ICOS*) rs4404254 T>C, rs10932029 T>C, *CD28* rs3116496 T>C and *CD80* rs7628626 C>A SNPs and assessed the potential relationship of these SNPs with hepatocellular carcinoma (HCC) risk. A total of 584 HCC cases and 923 healthy controls were recruited. And SNPscan™ genotyping assay was used to obtain the genotypes of *ICOS, CD28* and *CD80* polymorphisms. We found that *ICOS* rs10932029 T>C polymorphism significantly increased the risk of HCC (additive model: adjusted odds ratio (OR), 1.59; 95% confidence interval (CI), 1.13–2.22; *P*=0.007; homozygote model: adjusted OR, 1.12; 95% CI, 0.31–4.03; *P*=0.867; dominant model: adjusted OR, 1.58; 95% CI, 1.14–2.19; *P*=0.007 and recessive model: adjusted OR, 1.02; 95% CI, 0.28–3.68; *P*=0.974). However, *ICOS* rs4404254 T>C, CD28 rs3116496 T>C and CD80 rs7628626 C>A SNPs were not associated with the risk of HCC. To evaluate the effects of *ICOS* rs10932029 T>C on HCC risk according to different age, gender, chronic hepatitis B virus (HBV) infection, tobacco consumption and drinking status, we carried out a stratification analysis. We found that *ICOS* rs10932029 T>C polymorphism might increase the risk of HCC in male, ≥53 years, never smoking, never drinking and non-chronic HBV infection subgroups. Our study highlights that *ICOS* rs10932029 T>C polymorphism may confer the susceptibility to HCC. It may be beneficial to explore the relationship between variants in immune related genes and the development of HCC.

## Introduction

Hepatocellular carcinoma (HCC) remains a major public health problem worldwide, especially in China. [1] The etiology of HCC is very complicated. It is reported that many environmental factors and unhealthy lifestyles may influence the development and progress of HCC. The potential risk factors contributing to HCC are chronic hepatitis B virus (HBV) infection, aflatoxin, foods preserved by salting, smoking and drinking et al. [2–4] Although a growing number of investigations have focused on the etiology of HCC, it is not fully understood. It is suggested that an individual’s hereditary factor is also implicated in pathogenesis of HCC. Recently, a number of studies reported that some immune related gene variants might play important roles in the development of HCC [5–7].

The process of T-cell activity is very complex. Several transmembrane receptor/ligand pairs cooperate with the T-cell receptor to inhibit or enhance the activity of T cells [8]. The CD28 immunoglobulin superfamily involves the co-inhibitory molecules CTLA-4 and PD-1 as well as the costimulatory molecules inducible T-cell costimulator (ICOS) and CD28. ICOS gene shares homology with human *CD28* gene [9]. Recently, it has been identified that ICOS may be up-regulated along with T-lymphocyte activation and then interacts with its ligand (ICOSL). Finally, these processes promote T-lymphocyte proliferation and T helper 2 (Th2) differentiation [10]. Nagase et al. [11] reported that ICOS+Foxp3+ tumor infiltrating lymphocytes were associated with prognosis of gastric cancer and effector regulatory T cell (Treg) correlated with *Helicobacter pylori*. In addition, a previous study suggested that Treg, especially ICOS^+^Foxp3^+^Treg, might be increased in the HCC microenvironment and predict reduced survival [12]. Based on the vital roles of participation in both T-lymphocyte proliferation and Th2 differentiation, any variant of *ICOS* gene may influence the development and carcinogenesis of HCC. The *ICOS* gene is polymorphic, which is located on chromosome 2 in humans. Several *ICOS* polymorphisms [e.g. rs10932029 T>C, rs4404254 T>C, rs4675379 G>C, rs10932037 C>T (ISV1+173T>C) and rs10183087 A>C] have been established. Among these single nucleotide polymorphisms (SNPs), *ICOS* rs10932029 T>C and rs4404254 T>C were most widely studied for their susceptibility to various cancers [13–16]. However, the observed results remain inconsistent rather than conclusive.

CD28 is expressed by most T cells, which competes with CTLA-4 for B7 binding and promotes T-cell proliferation. Recently, some epidemiological studies indicated the potential relationship between *CD28* rs3116496 T>C (IVS3 +17T>C) variants and cancer susceptibility. Several publications reported that *CD28* rs3116496 TT genotype conferred a low penetrance risk to breast cancer and cervical cancer [17,18]. However, the association between *CD28* rs3116496 T>C (IVS3 +17T>C) variants and HCC risk remains unknown.

CD80 (also B7-1) is a protein expressed on activated B cells, dendritic cells and monocytes, which provides a costimulatory signal for T-lymphocyte activation and survival. It is the ligand for CD28 (for auto-regulation and intercellular association). Wu et al. [13] reported that *CD80* rs7628626 C>A variants were not associated with the risk of CRC; however, *CD80* rs7628626 C>A variants were closely related to regional lymph node metastasis and aggressive tumor progression. Thus, *CD80* rs7628626 C>A may be implicated in the development of cancer.

Here, we selected *ICOS* rs4404254 T>C, rs10932029 T>C, *CD28* rs3116496 T>C and *CD80* rs7628626 C>A polymorphisms and carried out a hospital-based case–control study to explore the potential association of *ICOS, CD28* and *CD80* SNPs with the risk of HCC.

## Materials and methods

### Subjects

A total of 584 cases with HCC and non-cancer controls (*n*=923) were recruited. HCC cases were enrolled in Fuzong Clinical Medical College and Union Clinical Medical College of Fujian Medical University, Fuzhou, China. Controls were included voluntarily, who participated in a routine medical check-up. All participants were eastern Chinese Han population and unrelated. HCC patients underwent operation, and the pathological findings were confirmed by two experienced pathologists. Controls were fully matched with HCC cases in terms of sex and age. Each participant signed a written informed consent. Risk factors (smoking and drinking) and demographic variables were collected by an interview. Hepatitis B surface antigen (HBsAg) was measured. The criteria of ‘smoker’ and ‘drinker’ were described in the previous study [19]. The corresponding data are presented in Table 1. The whole blood was donated by each participant and stored immediately at −80°C until use. The study protocol was approved by Institutional Review Board at Fujian Medical University.

**Table 1:** Distribution of selected demographic variables and risk factors in HCC cases and controls

Variable	HCC cases (*n*=584)		Healthy controls (*n*=923)		*P*^1^
	*n*	%	*n*	%	
Age (years)	53.17 (±11.76)		53.72 (±9.97)		0.327
Age (years)					0.358
<53	264	45.21	395	42.80	
≥53	320	54.79	528	57.20	
Sex					0.717
Male	525	89.90	835	90.47	
Female	59	10.10	88	9.53	
Smoking status					0.834
Never	374	64.04	596	64.57	
Ever	210	35.96	327	35.43	
Alcohol use					**<0.001**
Never	414	70.89	775	83.97	
Ever	170	29.11	148	16.03	
Chronic HBV infection					**<0.001**
Yes	412	70.55	85	9.21	
No	172	29.45	838	90.79	

Bold values are statistically significant (*P*<0.05).

1 Two-sided *χ*^2^ test and Student’s *t* test.

### Selection of SNPs

The polymorphisms of *ICOS, CD28* and *CD80* gene were selected based on publications, [13–18] in which polymorphisms were studied the association with the risk of cancer. Finally, *ICOS* rs4404254 T>C, rs10932029 T>C, *CD28* rs3116496 T>C and *CD80* rs7628626 C>A were selected and studied. The primary information of *ICOS* rs4404254 T>C, rs10932029 T>C, *CD28* rs3116496 T>C and *CD80* rs7628626 C>A SNPs is summarized in Table 2.

**Table 2:** Primary information for *ICOS* rs4404254 T>C, rs10932029 T>C, *CD28* rs3116496 T>C and *CD80* rs7628626 C>A SNPs

Genotyped SNPs	Chromosome	Chr. Pos. (NCBI Build 38)	Region	MAF^1^ for Chinese in database	MAF in our controls (*n*=923)	*P-*value for HWE^2^ test in our controls	Genotyping method	Genotyping value (%)
*ICOS* rs10932029 T>C	2	203937045	Intron	0.08	0.09	0.962	SNPscan	99.27
*ICOS* rs4404254 T>C	2	203960563	3′UTR	0.13	0.17	0.442	SNPscan	99.27
*CD28* rs3116496 T>C	2	203729789	Intron	0.10	0.10	0.821	SNPscan	99.27
*CD80* rs7628626 C>A	3	119525574	3′UTR	0.12	0.12	0.948	SNPscan	99.27

1 MAF, minor allele frequency.

2 HWE, Hardy–Weinberg equilibrium.

### DNA extraction and genotyping

Using the DNA Purification Kit (Promega, Madison, U.S.A.), we extracted the genomic DNA from lymphocytes. The obtained DNA was stored at −80°C until use. The concentration and purity were measured by micro-spectrophotometer. SNPscan™ genotyping assay (Genesky Biotechologies Inc., Shanghai, China), a double ligation and multiplex fluorescence PCR, [20] was used to analyze the variants of *ICOS* rs4404254 T>C, rs10932029 T>C, *CD28* rs3116496 T>C and *CD80* rs7628626 C>A polymorphisms. The success rates of *ICOS* rs4404254 T>C, rs10932029 T>C, *CD28* rs3116496 T>C and *CD80* rs7628626 C>A genotyping are shown in Table 2. For quality control, four percent of overall DNA samples were randomly selected and analyzed. And the reproducibility was 100%.

### Statistical analysis

Age of participants was described as the mean ± standard deviation (SD). And Student’s *t* test was used to compare the difference among the HCC cases and non-cancer controls. An online software (http://ihg.gsf.de/cgi-bin/hw/hwa1.pl) was used to measure whether genotype distributions of *ICOS* rs4404254 T>C, rs10932029 T>C, *CD28* rs3116496 T>C and *CD80* rs7628626 C>A in controls deviate from Hardy–Weinberg equilibrium (HWE) [19,21–27]. Chi-square test (*χ*^2^) or Fisher exact test was harnessed to compare the categorical variables (e.g. frequencies of *ICOS* rs4404254 T>C, rs10932029 T>C, *CD28* rs3116496 T>C and *CD80* rs7628626 C>A genotypes, age, sex, smoking status and drinking). Multivariate logistic regression was used to calculate the crude/adjusted odds ratios (ORs) and their 95% confidence intervals (CI) for the correlation of *ICOS* rs4404254 T>C, rs10932029 T>C, *CD28* rs3116496 T>C and *CD80* rs7628626 C>A polymorphisms with HCC susceptibility. We used SAS 9.4 software for Windows (SAS Institute Inc., Cary, NC, U.S.A.) to perform all statistical analysis. The statistical significance was considered as *P*<0.05 (two-tailed). Power and Sample Size online software (http://biostat.mc.vanderbilt.edu/twiki/bin/view/Main/PowerSampleSize) was used to obtain the value of power (α = 0.05) [28].

## Results

### Baseline characteristics

The information of demographics (age and sex) and selected susceptibility factors (status of chronic HBV infection, smoking and drinking) are summarized in Table 1. As demonstrated in Table 1, this case–control study was matched by age, sex and smoking status (*P*=0.327, *P*=0.717 and *P*=0.834 respectively). We found a significant difference in status of chronic HBV infection and drinking between the HCC patients and the controls (*P*<0.001). For *ICOS* rs4404254 T>C, rs10932029 T>C, *CD28* rs3116496 T>C and *CD80* rs7628626 C>A polymorphisms, the success rate of genotyping was more than 99.00% (Table 2). In our study, the minor allele frequencies (MAFs) of *ICOS* rs4404254 T>C, rs10932029 T>C, *CD28* rs3116496 T>C and *CD80* rs7628626 C>A were similar to the data for Chinese Han population. The distributions of *ICOS* rs4404254 T>C, rs10932029 T>C, *CD28* rs3116496 T>C and *CD80* rs7628626 C>A genotype frequencies were accorded with HWE (Table 2).

### Association of ICOS rs4404254 T>C, rs10932029 T>C, CD28 rs3116496 T>C and CD80 rs7628626 C>A polymorphisms with HCC

The genotype distributions of *ICOS* rs4404254 T>C, rs10932029 T>C, *CD28* rs3116496 T>C and *CD80* rs7628626 C>A variants are summarized in Table 3.

**Table 3:** The frequencies of *ICOS* rs4404254 T>C, rs10932029 T>C, *CD28* rs3116496 T>C and *CD80* rs7628626 C>A polymorphisms in HCC patients and controls

Genotype	Overall HCC case (*n*=584)		Overall controls (*n*=923)	
	*n*	%	*n*	%
*ICOS* rs10932029 T>C				
TT	420	73.04	756	82.08
TC	146	25.39	157	17.05
CC	9	1.57	8	0.86
CT+CC	155	26.96	165	17.92
TT+CT	566	98.43	913	99.13
C allele	164	14.26	173	9.39
*ICOS* rs4404254 T>C				
TT	383	66.61	642	69.71
TC	172	29.91	250	27.14
CC	20	3.48	29	3.15
CT+CC	192	33.39	279	30.29
TT+CT	555	96.52	892	96.85
C allele	212	18.43	308	16.72
*CD28* rs3116496 T>C				
TT	466	81.04	751	81.54
TC	99	17.22	162	17.59
CC	10	1.74	8	0.87
CT+CC	109	18.96	170	18.46
TT+CT	565	98.26	913	99.13
C allele	119	10.35	178	9.66
*CD80* rs7628626 C>A				
CC	445	77.39	721	78.28
CA	120	20.87	188	20.41
AA	10	1.74	12	1.30
CA+AA	130	22.61	200	21.72
CC+CA	565	98.26	909	98.70
A allele	140	12.17	212	11.51

The frequencies of *ICOS* rs10932029 TT, TC and CC genotypes were 73.04, 25.39 and 1.57% in 584 HCC patients and 82.08, 17.05, and 0.86% in 923 controls, respectively. When compared with the frequency of *ICOS* rs10932029 TT genotype, there was a significant difference in the frequency of *ICOS* rs10932029 TC genotype between the HCC patients and control subjects (crude OR = 1.64, 95% CI: 1.27–2.12, *P*<0.001). When the frequency of *ICOS* rs10932029 TT genotype was used as a reference, we found no difference in the frequency of *ICOS* rs10932029 CC genotype between the HCC patients and control subjects (crude OR = 1.99, 95% CI: 0.76–5.19, *P*=0.161). When compared with the frequency of *ICOS* rs10932029 TT genotype, there was a difference in the frequency of *ICOS* rs10932029 TC/CC genotype between HCC patients and the controls (crude OR = 1.69, 95% CI: 1.32–2.17, *P*<0.001). When the frequency of *ICOS* rs10932029 TT/TC genotype was used as reference, there was no difference in the frequency of *ICOS* rs10932029 CC genotype between HCC patients and the controls (crude OR = 1.82, 95% CI: 0.76–4.73, *P*=0.223). Adjustment for age, sex, chronic HBV infection, smoking and drinking, these potential associations were not altered (additive model: adjusted OR, 1.59; 95% CI, 1.13–2.22; *P*=0.007; homozygote model: adjusted OR, 1.12; 95% CI, 0.31–4.03; *P*=0.867; dominant model: adjusted OR, 1.58; 95% CI, 1.14–2.19; *P*=0.007 and recessive model: adjusted OR, 1.02; 95% CI, 0.28–3.68; *P*=0.974; Table 4).

**Table 4:** Overall analysis of *ICOS* rs4404254 T>C, rs10932029 T>C, *CD28* rs3116496 T>C and *CD80* rs7628626 C>A polymorphisms with HCC

Genotype	Overall (584 cases vs. 923 controls)			
	Crude OR (95% CI)	*P*	Adjusted OR^1^ (95% CI)	*P*
*ICOS* rs10932029 T>C				
Additive model	**1.64 (1.27–2.12)**	**<0.001**	**1.59 (1.13–2.22)**	**0.007**
Homozygote model	1.99 (0.76**–**5.19)	0.161	1.12 (0.31**–**4.03)	0.867
Dominant model	**1.69 (1.32–2.17)**	**<0.001**	**1.58 (1.14–2.19)**	**0.007**
Recessive model	1.82 (0.76**–**4.73)	0.223	1.02 (0.28**–**3.68)	0.974
*ICOS* rs4404254 T>C				
Additive model	1.13 (0.90**–**1.42)	0.299	0.94 (0.69**–**1.28)	0.698
Homozygote model	1.13 (0.63**–**2.03)	0.675	1.21 (0.56**–**2.61)	0.636
Dominant model	1.15 (0.92**–**1.44)	0.210	0.98 (0.73**–**1.31)	0.884
Recessive model	1.11 (0.62**–**1.98)	0.728	1.24 (0.58**–**2.66)	0.587
*CD28* rs3116496 T>C				
Additive model	0.97 (0.74**–**1.28)	0.821	0.87 (0.60**–**1.25)	0.437
Homozygote model	1.98 (0.78**–**5.06)	0.153	1.54 (0.44**–**5.44)	0.503
Dominant model	1.03 (0.79**–**1.35)	0.809	0.91 (0.64**–**1.29)	0.594
Recessive model	2.02 (0.79**–**5.15)	0.141	1.59 (0.45**–**5.61)	0.468
*CD80* rs7628626 C>A				
Additive model	1.02 (0.79**–**1.32)	0.901	1.00 (0.71**–**1.40)	0.998
Homozygote model	1.33 (0.57**–**3.10)	0.513	1.72 (0.57**–**5.19)	0.332
Dominant model	1.05 (0.82**–**-1.35)	0.684	1.05 (0.76**–**1.46)	0.777
Recessive model	1.34 (0.58**–**3.12)	0.497	1.73 (0.58**–**5.20)	0.326

1 Adjusted for age, sex, chronic HBV infection, smoking and alcohol use in a logistic regression model.

However, in our study, no significant association of *ICOS* rs4404254 T>C, *CD28* rs3116496 T>C and *CD80* rs7628626 C>A variants with the risk of HCC was found.

We used a software to calculate the power value (α = 0.05). For *ICOS* rs10932029 T>C, the power value was 0.940 in additive model and 0.942 in dominant model.

### Association of ICOS rs10932029 T>C polymorphism with HCC in different stratification groups

To evaluate the effects of *ICOS* rs10932029 T>C on HCC risk according to different age, gender, chronic HBV infection, smoking and drinking status, we carried out a subgroup analysis. Table 5 lists frequencies of *ICOS* rs10932029 T>C variants in the stratified analysis. After adjustment by logistic regression analysis with these risk factors, we found that *ICOS* rs10932029 T>C polymorphism might be associated with an increased risk of HCC in some subgroups [male group: TC vs. TT: adjusted OR = 1.47, 95% CI 1.01–2.12, *P*=0.043 and TC/CC vs. TT: adjusted OR = 1.49, 95% CI 1.04–2.13, *P*=0.031; ≥53 years subgroup: TC vs. TT: adjusted OR = 1.70, 95% CI 1.09–2.64, *P*=0.020 and TC/CC vs. TT: adjusted OR = 1.62, 95% CI 1.05–2.49, *P*=0.029; never smoking group: TC/CC vs. TT: adjusted OR = 1.49, 95% CI 1.00–2.22, *P*=0.049 and never drinking group: TC vs. TT: adjusted OR = 1.56, 95% CI 1.07–2.26, *P*=0.020 and TC/CC vs. TT: adjusted OR = 1.57, 95% CI 1.09–2.26, *P*=0.016 and non-chronic HBV infection group: TC vs. TT: adjusted OR = 1.85, 95% CI 1.25–2.73, *P*=0.002 and TC/CC vs. TT: adjusted OR = 1.81, 95% CI 1.23–2.66, *P*=0.003 (Table 5)].

**Table 5:** Stratified analyses between *ICOS* rs10932029 T>C polymorphism and HCC risk

Variable	*ICOS* rs10932029 T>C (case/control)^1^			Adjusted OR^2^ (95% CI); *P*				
	TT	TC	CC	TT	TC	CC	TC/CC	CC vs. (TC/TT)
Sex								
Male	379/683	129/143	9/7	1.00	**1.47 (1.01–2.12); *P*: 0.043**	1.56 (0.40**–**6.18); *P*: 0.525	**1.49 (1.04–2.13); *P*: 0.031**	1.45 (0.37**–**5.73); *P*: 0.595
Female	41/73	17/14	0/1	1.00	2.39 (0.94**–**6.05); *P*: 0.067	-	2.14 (0.86**–**5.29); *P*: 0.101	-
Age (years)								
<53	197/319	61/72	2/2	1.00	1.41 (0.85**–**2.35); *P*: 0.182	3.36 (0.34**–**33.23); *P*: 0.301	1.48 (0.89**–**2.44); *P*: 0.128	3.15 (0.32**–**31.09);*P*: 0.325
≥53	223/437	85/85	7/6	1.00	**1.70 (1.09–2.64); *P*: 0.020**	0.76 (0.18**–**3.18); *P*: 0.709	**1.62 (1.05–2.49); *P*: 0.029**	0.69 (0.17**–**2.85); *P*: 0.603
Smoking status								
Never	271/487	93/102	4/5	1.00	1.50 (1.00**–**2.24); *P*: 0.050	0.97 (0.17**–**5.49); *P*: 0.973	**1.49 (1.00–2.22); *P*: 0.049**	0.90 (0.16**–**5.08); *P*: 0.907
Ever	149/269	53/55	5/3	1.00	1.77 (0.95**–**3.31); *P*: 0.072	1.33 (0.19**–**9.37); *P*: 0.774	1.75 (0.95**–**3.20); *P*: 0.071	1.18 (0.17**–**8.21); *P*: 0.866
Alcohol consumption								
Never	299/635	103/132	6/6	1.00	**1.56 (1.07–2.26); *P*: 0.020**	1.41 (0.34**–**5.89); *P*: 0.640	**1.57 (1.09–2.26); *P*: 0.016**	1.30 (0.31**–**5.41); *P*: 0.721
Ever	121/121	43/25	3/2	1.00	1.61 (0.76**–**3.43); *P*: 0.218	0.33 (0.01**–**8.03); *P*: 0.497	1.50 (0.71**–**3.16); *P*: 0.285	0.30 (0.01**–**7.01); *P*: 0.451
Chronic HBV infection								
Yes	296/65	100/19	8/1	1.00	1.08 (0.60**–**1.95); *P*: 0.794	1.66 (0.19**–**14.92); *P*: 0.650	1.14 (0.64**–**2.03); *P*: 0.657	1.67 (0.19**–**14.96);*P*: 0.645
No	124/691	46/138	1/7	1.00	**1.85 (1.25–2.73); *P*: 0.002**	0.80 (0.10**–**6.60); *P*: 0.832	**1.81 (1.23–2.66); *P*: 0.003**	0.70 (0.08**–**5.79);*P*: 0.740

1 The genotyping was successful in 575 (98.46%) HCC cases and 921 (99.78%) controls for *ICOS* rs10932029 T>C.

2 Adjusted for age, sex, chronic HBV infection, smoking and alcohol consumption (besides stratified factors accordingly) in a logistic regression model.

## Discussion

HBV is considered as an important risk factor in the development of HCC. However, the incidence of HCC alters materially between similarly chronic HBV infection subjects, suggesting that hereditary factor may contribute to its development. Of late, a number of studies reported that immune related gene variants might be associated with the development of HCC [29–33]. In consideration of the role of *ICOS, CD28* and *CD80* genes in tumor immunity, we chose *ICOS* rs4404254 T>C, rs10932029 T>C, *CD28* rs3116496 T>C and *CD80* rs7628626 C>A SNPs to explore their potential roles in the etiology of HCC. In this case–control study, we found that *ICOS* rs10932029 T>C polymorphism was associated with the risk of HCC. In the stratified analysis, we found that *ICOS* rs10932029 T>C polymorphism might be associated with the risk of HCC in male, ≥53 years, never smoking, never drinking and non-chronic HBV infection subgroups.

Rs10932029 T>C polymorphism is located on first intron region of *ICOS* gene, [16] where a number of splicing and regulatory components may interact with it [34]. Recently, several case–control studies have assessed the relationship of *ICOS* rs10932029 T>C polymorphism with cancer risk [15,16,35,36]; however, the results are controversial. Several epidemiological studies reported that *ICOS* rs10932029 T>C polymorphism was not associated with the risk of cancer [16,35,36]. However, Xu et al. [15] found that compared with *ICOS* rs10932029 TT genotype and T allele, the *ICOS* rs10932029 CT genotype and C allele conferred a significantly increased susceptibility to breast cancer, and this correlation was also identified in a validation cohort. In addition, a previous study indicated that compared with *ICOS* rs10932029 TT genotype, *ICOS* rs10932029 CT genotype was associated with a higher rate of disease progression in B-cell chronic lymphocytic leukemia patients [35]. In this case–control study, we found *ICOS* rs10932029 T>C locus might be associated with an increased risk of HCC, which was similar to the results of the previous study [15]. In the future, the potential role of *ICOS* rs10932029 T>C on influencing the expression of ICOS in HCC patient blood cells should be assessed to support our findings.

There are some limitations that should be acknowledged. First, all participants were recruited in two local hospitals in Fuzhou City, China. These subjects might not fully represent the eastern Chinese Han population. Second, only four important SNPs in *ICOS, CD28* and *CD80* genes were selected, which lack sufficient power to assess the total inherited risk in these genes. In the future, a tagging or a fine-mapping study is needed to further explore the potential association between SNPs in *ICOS, CD28* and *CD80* gene and the development of HCC. Third, in the present study, there is lack of the data about the expression or function of ICOS associated with rs10932029 T>C polymorphism. Finally, for lack of information for co-variates (e.g. body mass index, diet, lifestyle and so on), a more precise assessment was not carried out.

In summary, our study highlights that *ICOS* rs10932029 T>C polymorphism was associated with the susceptibility of HCC, especially in male, ≥53 years, never smoking, never drinking and non-chronic HBV infection subgroups. Our primary study shows that immune related gene variants may be advantageous for exploring susceptible to HCC.

## Abbreviations

CI: confidence interval
CTLA-4: cytotoxic T-lymphocyte antigen 4
Foxp3: forkhead box p3
HBV: hepatitis B virus
HCC: hepatocellular carcinoma
HWE: Hardy–Weinberg equilibrium
ICOS: inducible T cell costimulator
OR: odds ratio
PD-1: programmed death-1
SNP: single nucleotide polymorphism
Th2: T helper 2
Treg: regulatory T cell
